# Association between prenatal vitamin D deficiency with dental caries in infants and children: a systematic review and meta-analysis

**DOI:** 10.1186/s12884-024-06477-0

**Published:** 2024-04-08

**Authors:** Mansour Bahardoust, Salar Salari, Nader Ghotbi, Elham Rahimpour, Meisam Haghmoradi, Homan Alipour, Mahsa Soleimani

**Affiliations:** 1 Department of Epidemiology, School of Public Health, Shahid Beheshti University of Medical Sciences, Tehran, Iran; 2 General Dentist, School of Dentistry, Isfahan Azad University, Isfahan, Iran; 3 Shiraz University of Medical Sciences, Shiraz, Iran; 4 Department of Orthopedic Surgery, Urmia University of Medical Sciences, Urmia, Iran; 5 Tabriz University of Medical Sciences, Tabriz, Iran; 6 School of Medicine, Iran University of Medical Sciences, Tehran, Iran

**Keywords:** Prenatal, Vitamin D deficiency, Dental caries, Children

## Abstract

Prenatal vitamin D (PVD) is a vital micronutrient for dental caries (DCs). The association between prenatal vitamin D deficiencies (PVDD) and DCs in children has been conflicting in different reports. This meta-analysis aimed to investigate the association between PVDD and DCs in children for the first time. We searched PubMed, Scopus, Web of Sciences, Embase, and Scholar databases to find relevant studies based on mesh terms from 2000 to October 2023. This study was conducted based on the 2020 version of the PRISMA checklist. Cochran’s Q and I2 tests were used to evaluate heterogeneity between studies. Egger’s test was used to evaluate publication bias. The effect size of the association between PVDD and DCs was reported by the odds ratio (OR) at the 95% confidence interval (95% CI).Twelve studies, including 11,021 participants, were reviewed. The pooled prevalence of PVDD was estimated at 4353 (32%). The prevalence of DCs in children of mothers with and without PVDD was 44% and 25%, respectively. PVDD was significantly associated with an increased risk of DCs in children (OR: 1.35, 95% CI (1.22, 1.47), I2 = 86.6%). The association of DCs with PVDD was different based on gestational age groups, children’s age groups, and vitamin D levels. This meta-analysis showed PVDD can be associated with an increased risk of DCs in children, especially in mothers with prenatal vitamin D levels ≤ 35 nmol/L. Adequate vitamin D levels throughout pregnancy can help prevent DCs in children.

**Supplementary Information:**

The online version contains supplementary material available at 10.1186/s12884-024-06477-0.

## Background

Dental caries (DCs) are one of the common chronic disorders that can lead to pain, chewing problems, and discomfort [1–3]. As a result, it can negatively affect the quality of life of affected people, especially in the first year of life, and sometimes these effects can last for many years [4]. Tooth decay in children under 8 years of age is a multifactorial disease, and various factors such as physical, genetic, biological, environmental, and behavioral factors can play a role in its development [5–12].

The role of nutrition and micronutrients in maintaining oral and dental health is undeniable [13]. Among these nutrients, vitamin D has been widely considered one of the most important micronutrients due to the fundamental defect in bone health and maintaining the health and integrity of the tooth structure [14, 15]. In developed countries, DCs are estimated to affect 60–90% of pediatrics and school-age children [13]. Studies have shown that the formation of human teeth takes place in a unique period [16]. The structure and metabolism of teeth become relatively stable after mineralization [16]. Since the formation and mineralization of deciduous teeth begin during pregnancy [15, 17]. Therefore, the intrauterine environment, which during pregnancy is influenced by factors such as smoking and the mother’s nutrition, can significantly affect the growth, development, strength, mineralization of teeth, and tooth decay [17, 18].

Vitamin D status based on the predominant form of plasma vitamin D is clinically measured [19, 20]. Because the concentration of vitamin D in the fetus depends on the concentration of this vitamin in the mother, achieving an optimal and normal concentration level of 25(OH) D during the pregnancy is very important [19]. A different definition of the optimal concentration has been stated; many studies have defined a level ≥ 75 nmol/L as the optimal concentration [21]. While the Institute of Medicine (IOM) has defined ≥ 50 nmol/L as an adequate level [22]. According to some studies, there are several biologically plausible ways in which vitamin D may increase a child’s risk of caries [23, 24].It has been suggested that the formation of the developing tooth bud is influenced by serum 25(OH)D [25]. Enamel hypoplasia is a known risk factor for DC and it may result from prenatal vitamin D deficiency during primary tooth enamel formation periods. Additionally, decreased levels of cathelicidin and defensins, which are antimicrobial peptides, may lower the immune response [25]. These peptides attack cariogenic bacteria, reducing the risk of developing DCs [24]. Modifying the composition and flow of saliva could be an alternative approach, which may lead to a reduced concentration of calcium ions in saliva, according to a study [26].

Contradictory results regarding the association between PVDD and DCs in infants and children have been reported. C Suárez-Calleja et al [27] reported that vitamin D levels in mothers during pregnancy were significantly associated with DCs in children. K Tanaka et al. [4] showed that maternal vitamin D deficiency during pregnancy may be associated with an increased risk of DCs in children. In contrast, RJ Schroth [28] did not report a significant relationship between vitamin D levels in mothers during pregnancy and DCs in children.

Therefore, in this meta-analysis, for the first time, we investigated the association of prenatal vitamin D deficiencies (PVDD) with DCs in infants and children. The study can help clarify the association between vitamin D concentration during pregnancy and DCs in children with a comprehensive estimate. The results of this study can help health policymakers make decisions about interventions during pregnancy to prevent DCs in children.

## Methods

In this meta-analysis, we evaluated all original studies investigating the association of PVDD with DCs in children and infants. PubMed, Scopus, Web of Sciences, Embase, and Scholar databases were searched for relevant studies from 2000 to October 2023. The study was conducted using the PRISMA (PRISMA) version 2020 checklist [29]. 

### Literature search, screening, and data entry

First, the search strategy was determined for different search strategy databases. Two independent researchers (MB) and (FG) searched for related studies based on terms. The last search was done on October 5, 2023. The research question was defined based on PICO (Population, Intervention, Comparison, and Outcomes (.Population, intervention and outcome were defined as infants and children,) PVDD and DCs, respectively. The general search strategy was defined as follows.

(“Vitamin D Deficiency” OR " Vitamin D " OR “25-hydroxyvitamin” OR “25-hydroxyvitamin D” OR hydroxycholecalciferols” OR “[25(OH)D]” OR” ergocalciferols OR dihydroxycholecalciferols” OR “25-hydroxyvitamin D2) AND (“Dental Caries” OR ‘’ Dental Cavity’’ OR “Dental Decay” OR " Dental Cavities” OR “oral health” OR “Carious Lesions” OR “dental diseases” OR “Carious Dentin” OR “Dental White Spot " OR " Dental White Spots " OR “carious) and (“Pregnancies” OR “Pregnancy” OR " Prenatal” OR " Gestation”) AND (“Infants” OR “Infant” OR “Children”).

**Fig. 1:**
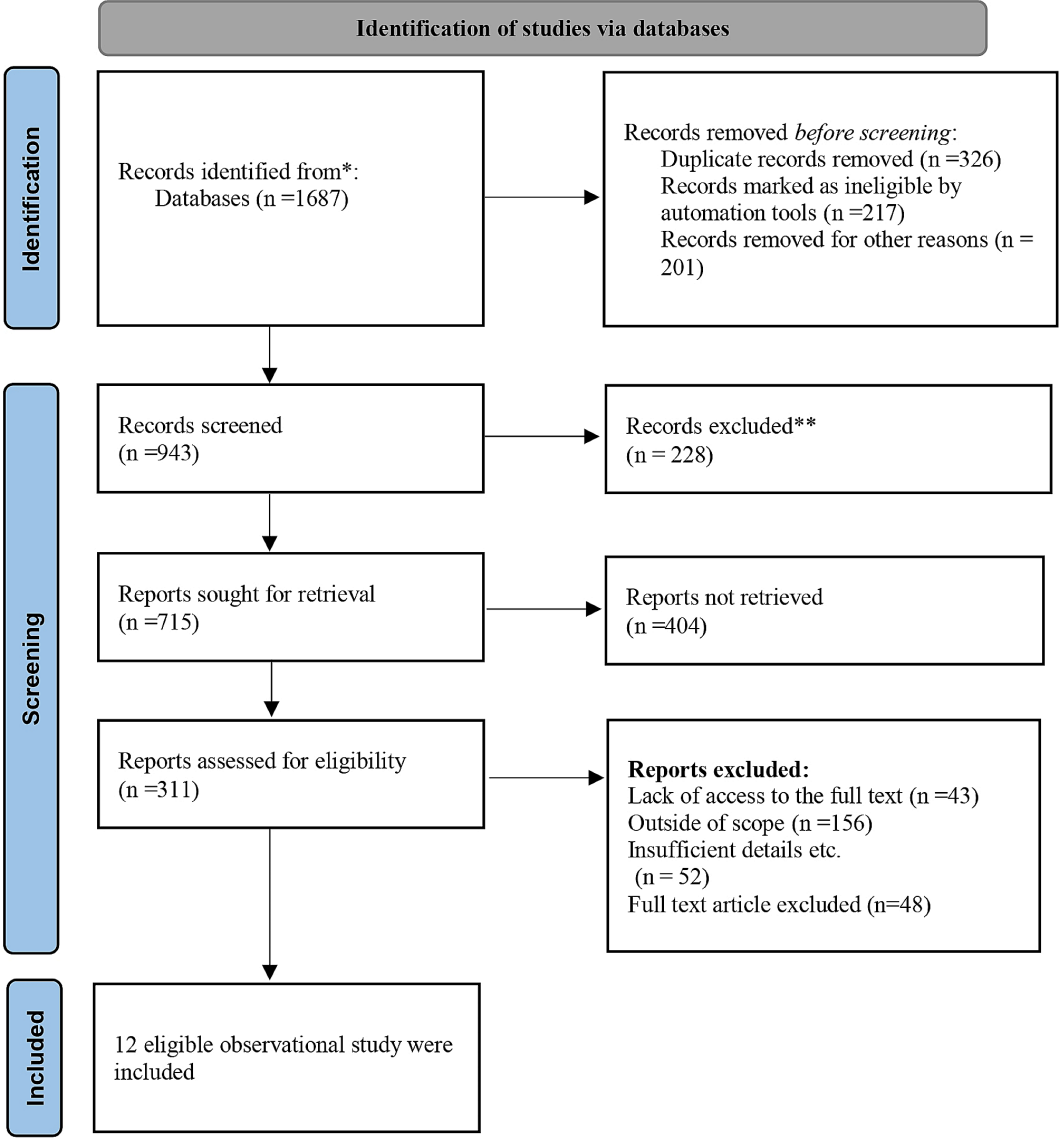
Flowchart page of studies based on PRISMA 2020

After searching, 1687 articles were extracted. Two independent researchers screened the studies based on the abstract and title using Endnote version 21 software. A number of 326 duplicate studies between the search databases were detected. Also, 418 studies (articles in non-English language, letters to the editor, case reports, and other reasons) were excluded from the study. The remaining 943 articles were examined using the title, objective, and abstract with the research question based on PICO. Six hundred thirty-two articles were excluded from the initial screening. The full text of 311 was studied in detail. Finally, 12 studies that investigated the relationship between PVDD and DCs were included (Fig. 1).

### Data collection

The research question and literature review determined the variables for this comprehensive review. The checklist for data collection was defined by experts in perinatologists, nutritionists, dentists, and epidemiologists. The variables of this study include authors, country, year of study, study design, number of all participants, number of mothers with vitamin D deficiency(< 35 nmol/L or < 50 nmol/L), number of mothers with a normal level of vitamin D, gestational age, age of children, number of DC in each group, children’s age groups, children’s sex, overall average level of prenatal vitamin D in mothers, Dt score in each group, history of vitamin D consumption during pregnancy, mothers’ education level(< 13 degree), maternal smoking during pregnancy, Effect size (odds ratio (OR) and 95% confidence interval (95% CI)) were extracted using Excel for articles.

### Eligibility criteria and data extraction

The inclusion criteria for the study included original human studies, investigating the relationship between PVDD and DCs in children aged 2 to 8 years, and access to the full text of the articles. Studies published in languages other than English, review articles and meta-analyses, laboratory or animal studies, and case reports were defined as exclusion criteria.

### Quality assessment

We used the Newcastle-Ottawa Quality Assessment Form for Cohort Studies checklist to assess the quality of the studies included in this review [30]. This checklist evaluates the quality of studies in three sections: Selection, Comparability, and Outcome/Exposure. And they give a score for each item. The range of scores for this checklist ranged from 0 to 9. The quality classification of the studies includes good (3 or 4 scores for the selection dimension and one or two stars for the comparability dimension and 2 or 3 stars for the outcome/exposure dimension), fair (2 scores for the selection dimension and one or two stars for the comparability dimension and 2 or 3 stars for the outcome/exposure dimension) and Poor (0 or 1 score for the selection dimension, 0 stars for the comparability dimension, and 0 or 1 star for the outcome/exposure dimension). Searching, screening, data extraction, study quality assessment and data analysis were done by two independent researchers. If there is a difference on a study or a variable, the difference was resolved with the participation of a third researcher.

### Statistical analysis

Stata version 17 was used for data analysis. Primary endpoints included the prevalence of DCs in women with and without vitamin D deficiency. The main measure of the effect size was prevalence (ratio of Children with DCs to the total Children and ratio of Women with vitamin D deficiency during pregnancy to total pregnant women). The correlation between DCs and PVDD was estimated based on the odds ratio (OR) in the 95% confidence range. (95% CI). The data were analyzed with the approach of random effect to control the effects of the sample size of the studies on the pooled estimate. The heterogeneity and inconsistency between studies were evaluated using Cochran’s Q and I2 tests. We used multivariate linear meta-regression models with mixed effect to discover the effective factors of communication. Estimates adjusted for gestational age, children’s age and vitamin D levels in mothers were estimated. Egger’s test was used to estimate the publication bias in the studies, and its results were presented with a funnel plot. Tables and figures were used for descriptive purposes. Sensitivity analysis was used to estimate the effect size of each study on the overall result, and the weight of each study was determined. Meta-regression was performed to control heterogeneity for the variables of children’s age, gestational age and vitamin D levels during pregnancy. Trim and fill analysis was used to resolve publication bias if publication bias was present. Association between DC and prenatal vitamin D was reported with OR in 95% CI.

## Results

In this met analysis, 12 observational studies (12 cohort [4, 5, 27, 28, 31–37] and one cross-sectional study [38]), including 11,021 participants (pregnant mothers and children), were included in the study. The mean age of mothers at the time of pregnancy was 26.1 years. Ten were cohort studies. The majority of studies were of good and moderate quality.(Supplement 1- Table 1) The age range of the investigated children was in the groups of 2 to 4, 4 to 6, and 6 to 8 years. The measurement of vitamin D levels in most studies was in the range of 24 to 38 weeks of pregnancy. The mean deciduous teeth (dt) score was reported in 6 studies. The mean dt score in children with PVDD and abnormal vitamin D mothers was 1.16 ± 2.54 and 1.42 ± 2.1, respectively. The comparison of vitamin D deficiency in most studies was based on < 35 nmol/L. Most studies were conducted in developed countries (Europe and America) (Table 1).

**Table 1:** Demographic characteristics of included studies

Author (Year)	Country	Study design	Sample size	N mother with (d3) deficiency	N child with DC	Mean Age of pregnant mothers	Sex child (Male)	prenatal (D3) deficiency definition (nmol/L)	Childs age group (years)	Maternal educational level < 13 grade	Gestational Age (weeks)	Quality of studies
RJ Schroth(2014) [5]	USA	prospective cohort	207	65	30	19	75	≤ 35	4 to 6	63	12 to 18	Fair
K Tanaka(2015) [4]	Japanese	prospective cohort	1210	605	154	31.6	571	≤ 35	2 to 4	82	12 to 18	Good
J Christensen(2016) [31, 32]	Canada	prospective cohort	175	85	26	23.05	84	≤ 50	4 to 6	NA	6 to 38	Fair
S Korun(2017)	Turkey	prospective cohort	50	31	28	20.1	23	≤ 35	4 to 6	28	24 to 36	Poor
R Singleton(2019) [33]	Canada	prospective cohort	78	23	13	26.1	41	≤ 35	2 to 4	NA	16 to 38	Poor
MJ Silva(2019) [34]	Australia	prospective cohort	345	NA	111	NA	163	≤ 35	6 to 8	699	24 to 36	Good
RJ Schroth(2020) [28]	Canada	prospective cohort	283	126	26	29.5	154	≤ 35	2 to 4	76	6 to 38	Good
C Suárez-Calleja(2021) [48]	Spain	prospective cohort	178	110	38	25.7	87	≤ 35	4 to 6	NA	18 to 24	Fair
CLA Navarro(2021) [36]	Netherland	Cross -sectional	5257	*1218 *1402	*589 *457	30.6	2634	≤ 35 ≤ 50	4 to 6	1748	18 to 38	Good
D Olczak-Kowalczyk(2021) [35]	Poland	prospective cohort	1638	734	328	25.9	NA	≤ 50	4 to 6	NA	24 to 36	Good
DM Beckett(2022) [38]	New Zealand	prospective cohort	78	12	24	25.9	NA	≤ 50	4 to 6	NA	24 to 36	Poor
RJ Singleton(2022)	USA	Retrospective cohorts	1522	438	NA	28.2	94	≤ 50	4 to 6	NA	24 to 36	Fair

### Prevalence of prenatal vitamin D deficiency

**Fig. 2:**
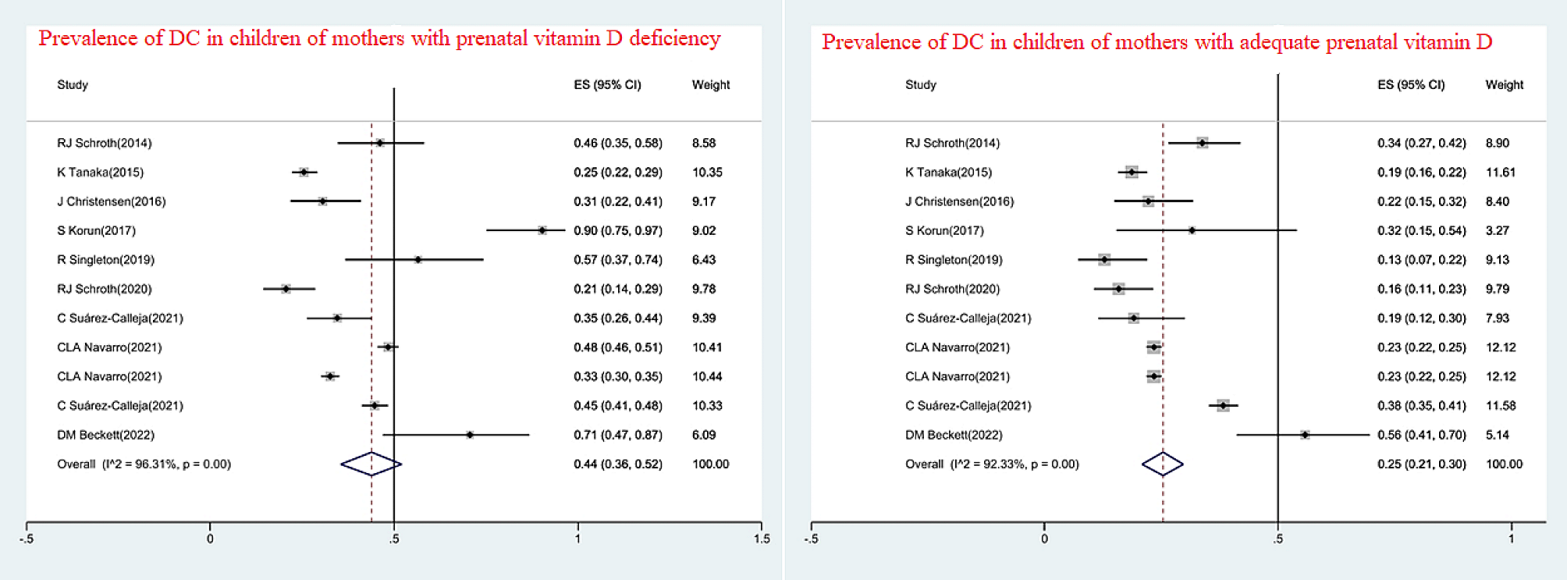
Prevalence of DC in children of mothers with prenatal vitamin D deficiency and mothers with adequate prenatal vitamin D

The pooled prevalence of PVDD was estimated at 4353(32%). The prevalence of DCs in children with mothers with low and sufficient prenatal vitamin D levels was 44% and 25%. (Fig. 2) According to the adjusted estimate of meta-regression, the prevalence of PVDD and DCs in children was 38% (95%CI: 30, 46%) and 21% (95%CI: 16, 26%), respectively.

## Prenatal vitamin D and children’s DC

**Fig. 3:**
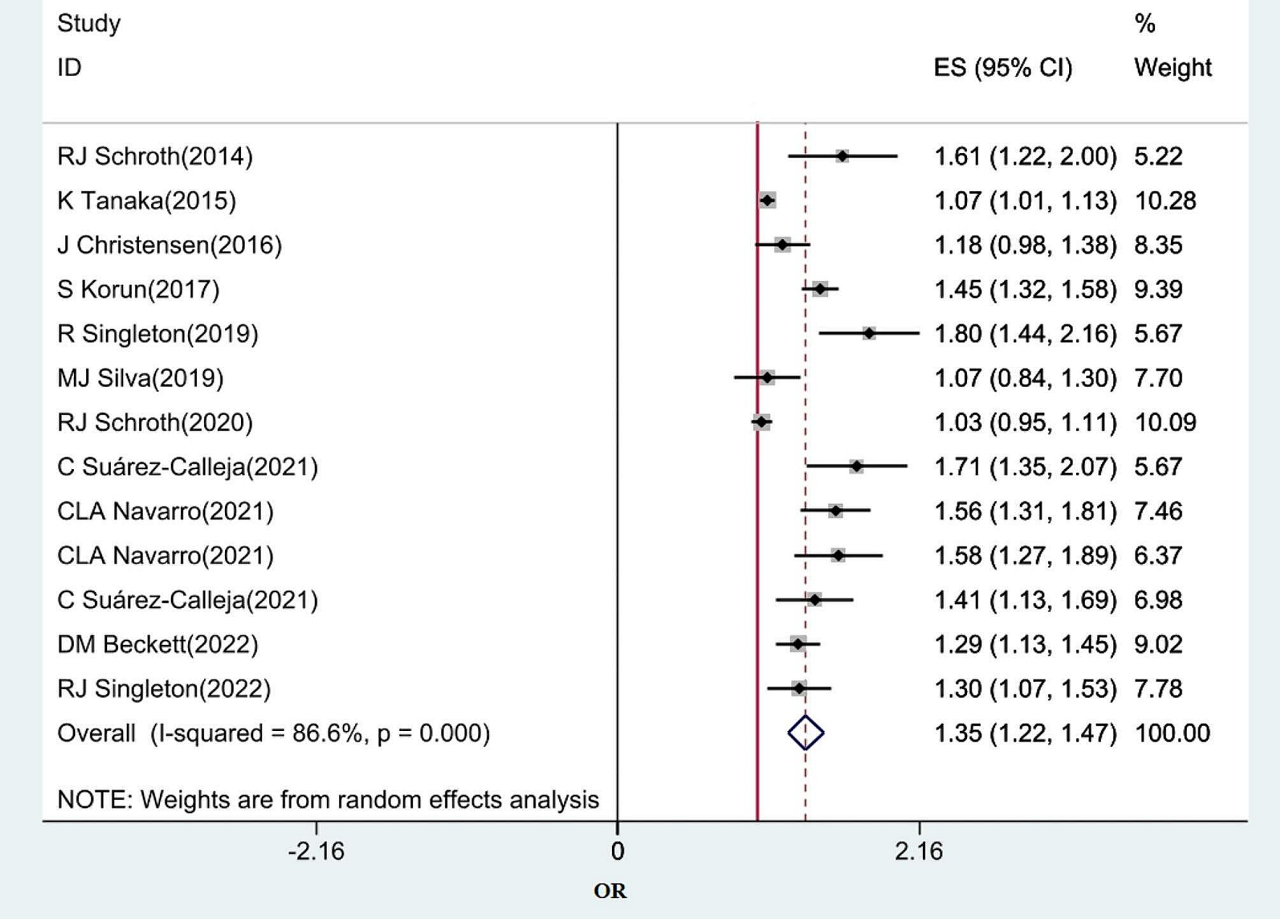
The association between prenatal vitamin D deficiency and children’s DC

**Fig. 4:**
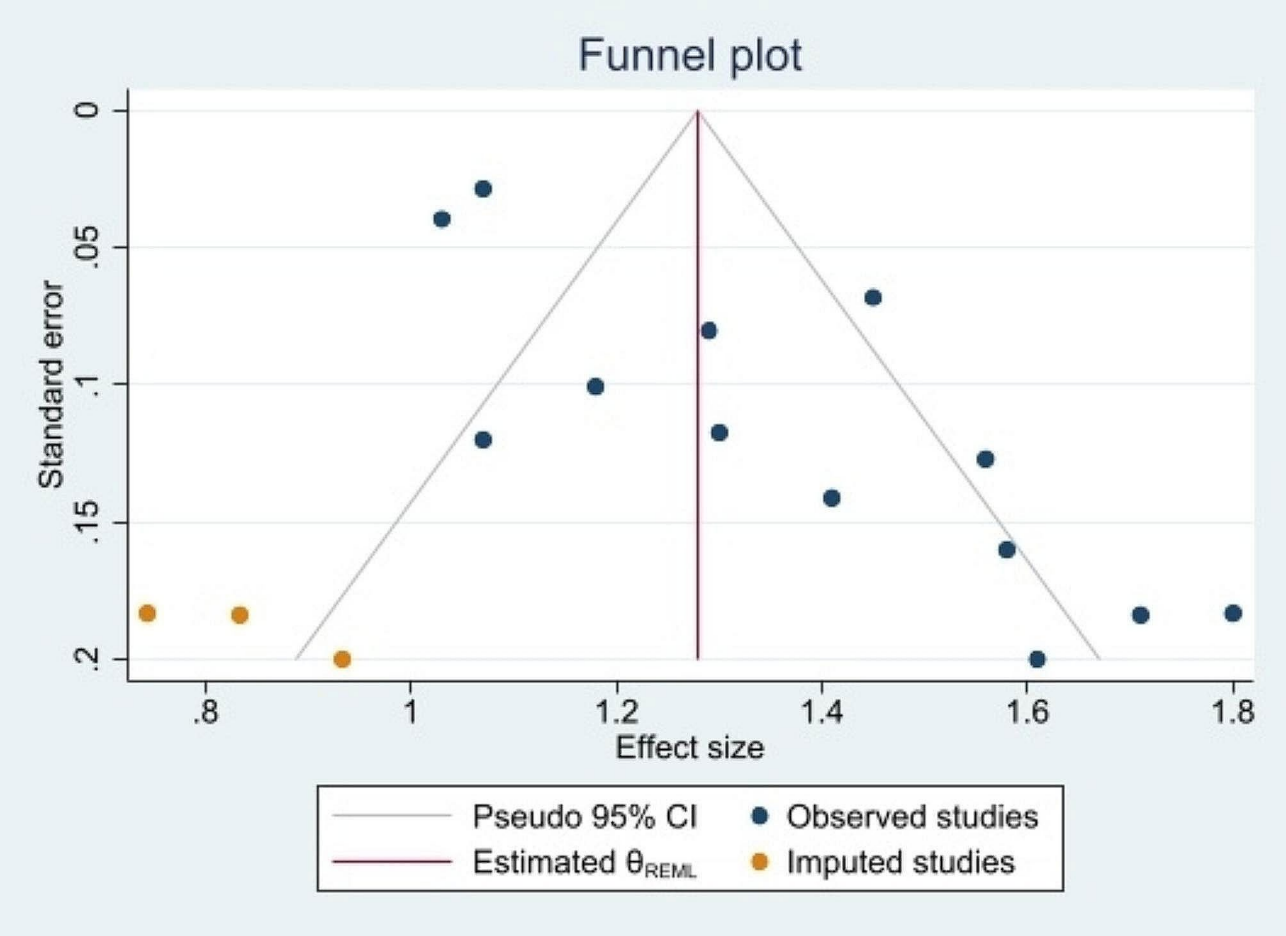
Bias publication assessment in the funnel plot

The pooled results of 13 studies showed that PVDD was significantly associated with an increased risk of DCs in children (OR: 1.35, 95% CI(1.22, 1.47), I^2^ = 86.6%). (Fig. 3) The adjusted estimate of meta-regression showed that DCs risk decreased by 1.28 (95% C:1.23,1.33) after adjusting for gestational age, children’s age, and mother’s vitamin D level. The results of the Egger test analysis showed that the publication bias creates negative results (Egger t test = 3.7, *P* = 0.001, 95% CI: 1.97, 5.32), which is shown as asymmetry in the funnel diagram. (Fig. 4). Due to significant publication bias, we used the Trim & Fill method to investigate the effect of censored studies on the pooled estimate. Our analysis showed that three studies appeared to be censored due to publication bias. After pooling their effect on the overall estimate, it was found that considering the unpublished articles, the pooled estimate of the OR decreased to 1.28 with a 95% CI of 1.14 to 1.48.

Sensitivity analysis was performed based on each study’s outcome, and each study’s effect on the overall estimate was determined.

### Sub-group analysis

**Fig. 5:**
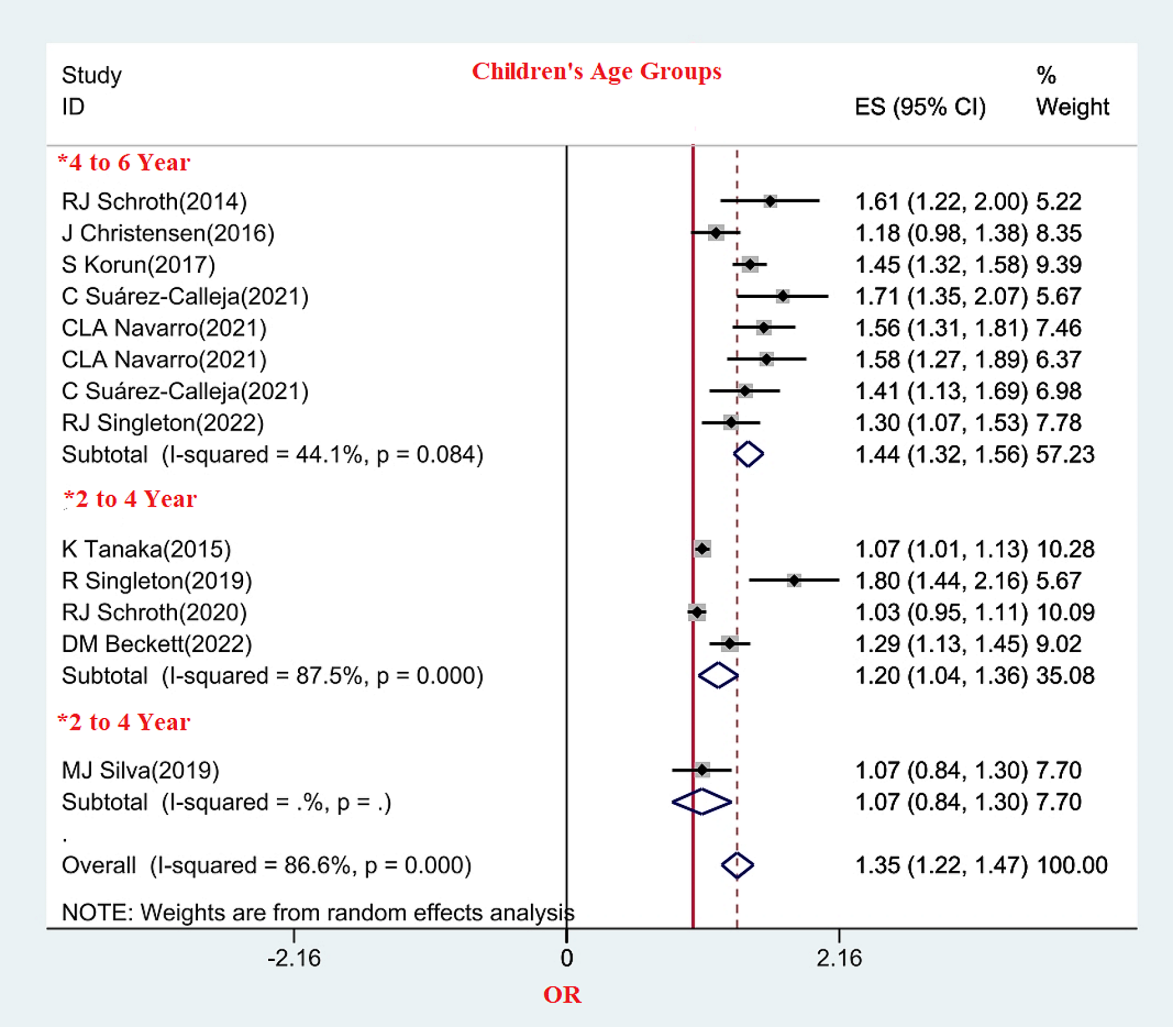
The relationship between prenatal vitamin D and DC in children’s age subgroups

Based on the results of age subgroup analysis, the pooled results of eight studies in children aged 4 to 6 years showed that the OR of DCs was significantly higher in this age group(OR: 1.44, 95% CI(1.32, 1.56), I^2^ = 44.1%). Only one study investigated the relationship between PVDD and DC in children aged 6 to 8 years, and this difference was not statistically significant(OR: 1.07, 95% CI(0.84, 1.30). (Fig. 5)

**Fig. 6:**
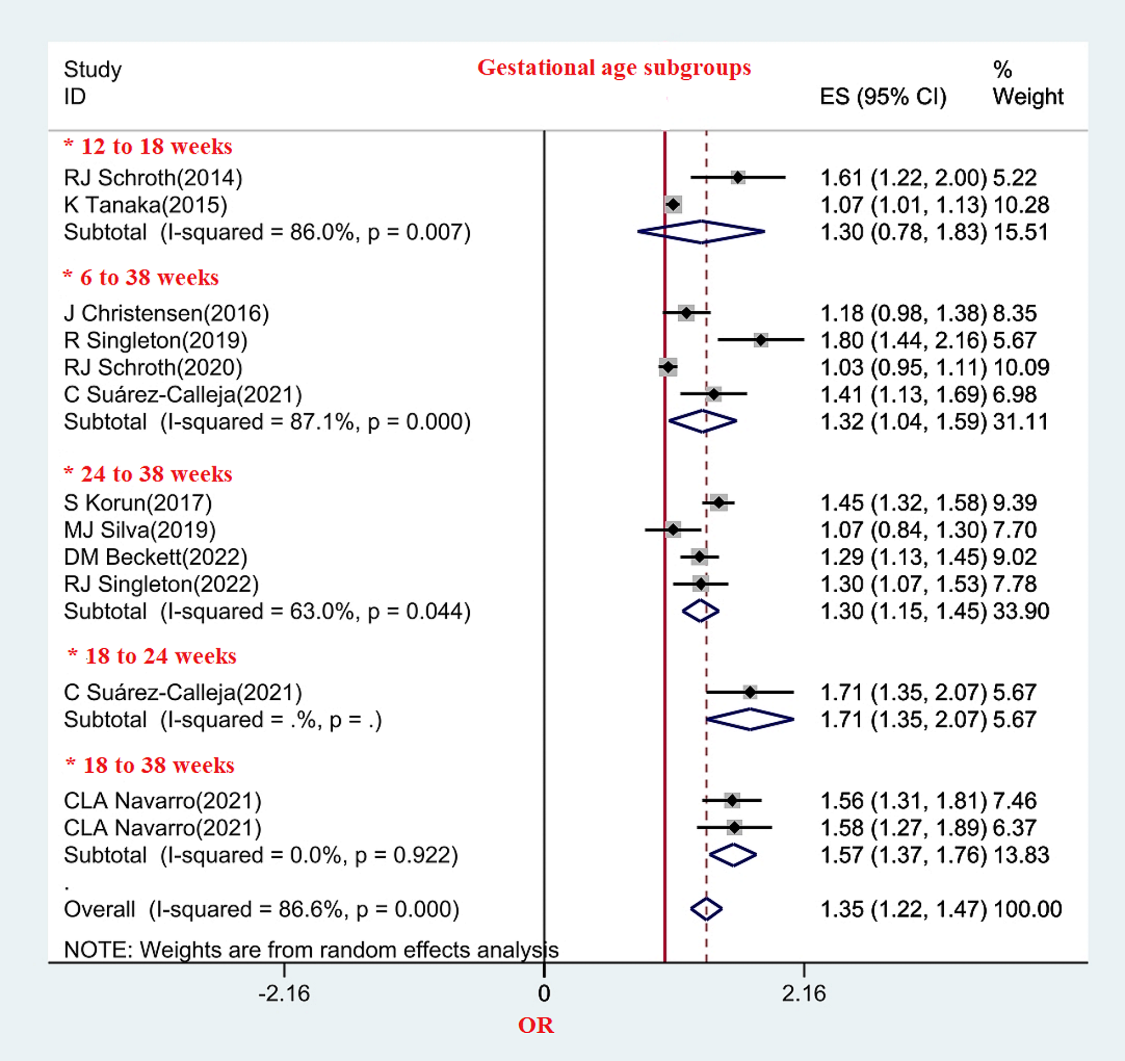
The relationship between prenatal vitamin D and DC in gestational age subgroups

The association of DCs in children with prenatal vitamin D deficiency was different in the gestational age groups of mothers. The association of DCs with prenatal vitamin D deficiency was investigated in 8 studies in weeks 6 to 38 and 24 to 38 of pregnancy, and the OR of DCs in children in these periods of pregnancy was 1.32 and 1.3, respectively. (Fig. 6)

**Fig. 7:**
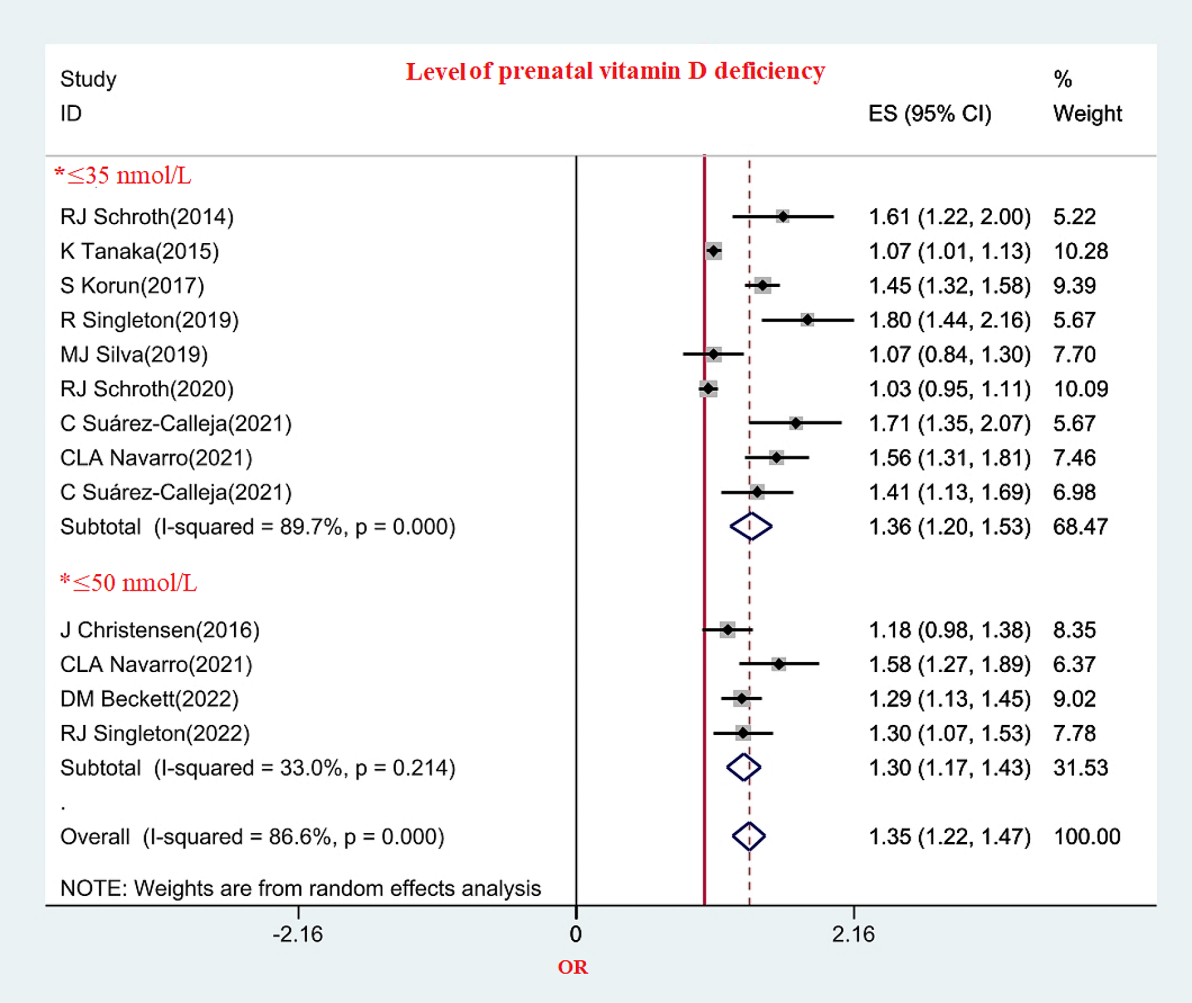
The relationship between prenatal vitamin D and DC based on level of vitamin D deficiency in different studies

The results of subgroup analysis based on PVDD showed that the OR of DCs in children with prenatal vitamin D levels ≤ 35 nmol/L was slightly higher than that of prenatal vitamin D levels ≤ 50 nmol/L. (Fig. 7)

## Discussion

According to our knowledge, there needs to be a clear, Comprehensive agreement on the role of PVDD with DCs in children, and conflicting results have been reported. A systematic review study has shown that deficiency of micronutrients and vitamins, especially vitamin D, is associated with oral health, tooth enamel defects, and tooth erosion in children [39, 40]. However, based on our knowledge, a comprehensive meta-analysis study has not investigated the relationship of prenatal vitamin D level with the risk of DCs in children based on various factors, including prenatal vitamin D level, gestational age, and children’s age. Therefore, for the first time in this meta-analysis study, by examining the results of 12 studies (11,021 participants), we evaluated the relationship between perinatal vitamin D levels and DC in children.

Our comprehensive review showed that the overall prevalence of vitamin D deficiency in pregnant women was nearly 32%. DC prevalence in children differed based on prenatal vitamin D levels. The prevalence of DC in children born to mothers with vitamin D deficiency during pregnancy was almost twice as high as in children with sufficient vitamin D levels. The pooled estimate showed that PVDD significantly increases the risk of DCs in all age groups under 8 years. The level of prenatal vitamin D had an inverse linear relationship with the prevalence of DCs in children, and increasing the level of prenatal vitamin D throughout pregnancy was associated with a decrease in the prevalence of DCs in children, especially between the ages of 4 and 6 years. The subgroup analysis showed that the increased risk of DCs was higher in children whose mothers had vitamin D levels ≤ 35 nmol/L during pregnancy than in mothers whose vitamin D levels were 35 to 50 or < 50 nmol/L. The highest prevalence of DCs was in children aged 4 to 6 years, and it seems that this age range is the peak incidence of DC in children, especially in children born to mothers with prenatal vitamin D deficiency. PVDD in the last weeks of pregnancy (> 24 weeks) or the third trimester of pregnancy may be associated with a further increase in the risk of DC in children. In a review study in 2023, MK Mahmood et al. [41] showed that the risk of DCs in children with low and poor serum vitamin D levels was significantly higher than in children with high serum vitamin D levels. Based on a dose-response analysis, their study showed an inverse linear relationship between serum vitamin D levels and the risk of DC. They reported that a 10 nmol/L increase in serum vitamin D levels in children was associated with a 3% significant reduction in DC risk. Our study also observed that prenatal vitamin D levels had an inverse linear relationship with the risk of DC in children; however, we could not perform a dose-response analysis. Our study and MK Mahmood et al. [41] show that vitamin D deficiency was associated with an increased risk of DCs during pregnancy in mothers and after birth in children.

In 2013, in a comprehensive review, G Tapalaga et al. [34] evaluated the role and effect of prenatal vitamin D on enamel defects and tooth erosion in 6978 participants. They showed that PVDD was associated with an increased risk of enamel defects, enamel hypoplasia, and DCs. It confirmed the results of our study. This result shows that vitamin D level before birth is significantly related to dental health in children, and special attention should be paid to adequate vitamin D levels during pregnancy. However, considering that the studies were conducted in different sample sizes and with different methodologies, the heterogeneity of the studies was not low, and stronger research should be done to determine the optimal level of vitamin D during pregnancy.

Mineral organs such as teeth are surrounded by alveolar bone. Tooth mineralization is dependent on the metabolism of minerals, so any disruption to this process can affect it [14, 15]. Vitamin D is one of the essential micronutrients that play a critical role in bone and tooth mineralization. A deficiency in vitamin D can lead to “rickets teeth,” making them more prone to fracture and decay [41–43]. Several mechanisms have been reported for the effect of vitamin D deficiency on tooth mineralization [43–45]. One of the primary biological mechanisms is that severe vitamin D deficiency causes hypocalcemia and hypophosphatemia with secondary hyperparathyroidism [46, 47]. This leads to increased bone turnover, which results in increased serum levels of calcium and low serum levels of mineralized phosphate [46, 47]. This prevents proper mineralization of teeth, making dentition susceptible to caries [42]. Based on the study quality assessment checklist results, most studies included in this regular review were of medium and good quality.

### Limitations

Our study had limitations that should be noted. Firstly, the heterogeneity of this review study was not low since the initial studies were conducted in different sample sizes and with different methodologies on different age groups of children and at different. However, the fact that after analyzing the results in subgroups and Doing meta-regression reduced this heterogeneity to an acceptable level, the heterogeneity of the studies can affect the overall estimate to some extent. Second, the primary studies were mostly conducted in developed countries and societies, and their results cannot be generalized to developing countries. Thirdly, in this study, although we tried to extract all the potential factors that could be related to the outcome from the primary studies, nevertheless, a number of important factors, such as vitamin D consumption during pregnancy by mothers, fluoride consumption in the child, exposure level Sunshine and seasons of birth, brushing and feeding, which can affect the results, were reported only in a limited number of studies, and missing data were high in these factors. Also, we only reviewed studies published in English in this systematic review.

Evaluating the association between PVDD and children’s DCs and examining this association based on different age groups of children and different gestational ages of mothers for the first time was the most important strength of our study.

## Conclusions

This systematic review showed that PVDD can be associated with an increased risk of DCs in children. The risk of DCs increased in lower levels of prenatal vitamin D compared to higher levels. Therefore, adequate vitamin D levels should be paid attention to throughout pregnancy as one of the important preventive strategies for DCs. PVDD in the last weeks of pregnancy (> 24 weeks) or the third trimester of pregnancy may be associated with a further increase in the risk of DC in children. However, due to the significant heterogeneity between the reviewed studies and the potential influence of intervening factors, more research is needed to confirm these findings in populations with different demographic characteristics.

### Electronic supplementary material

Below is the link to the electronic supplementary material.

Supplementary Material 1

## Data Availability

The datasets used and/or analysed during the current study are available from the corresponding author on reasonable request.
