# Supplementary material for: Association between prenatal vitamin D deficiency with dental caries in infants and children: a systematic review and meta-analysis
Source: BMC Pregnancy Childbirth. 2024 Apr 8;24:256. doi: 10.1186/s12884-024-06477-0 (PMC11000361; doi:10.1186/s12884-024-06477-0)
Supplement: Supplementary file 1 — Supplementary Material 1 [file 12884_2024_6477_MOESM1_ESM.docx]

**Table1: Quality assessment**

| Author | Selection | Comparability | Outcome/Exposure | Quality of studies |
| --- | --- | --- | --- | --- |
| RJ Schroth(2014) | ** | * | ** | Fair |
| K Tanaka(2015) | **** | ** | *** | Good |
| J Christensen(2016) | ** | * | ** | Fair |
| S Korun(2017) | * | * | * | Poor |
| R Singleton(2019) | ** | - | * | Poor |
| MJ Silva(2019) | *** | * | ** | Good |
| RJ Schroth(2020) | *** | * | ** | Good |
| C Suárez-Calleja(2021) | ** | * | *** | Fair |
| CLA Navarro(2021) | **** | ** | *** | Good |
| D Olczak-Kowalczyk(2021) | *** | ** | *** | Good |
| DM Beckett(2022) | * | * | * | Poor |
| RJ Singleton(2022) | *** | * | *** | Fair |
